# Translating Ultrasound into Clinical Practice for the Assessment of Swallowing and Laryngeal Function: A Speech and Language Pathology-Led Consensus Study

**DOI:** 10.1007/s00455-022-10413-9

**Published:** 2022-02-24

**Authors:** Jodi E. Allen, Gemma Clunie, Joan K.-Y. Ma, Margaret Coffey, Katharina Winiker, Sally Richmond, Soren Y. Lowell, Anna Volkmer

**Affiliations:** 1 The National Hospital for Neurology and Neurosurgery, Therapy & Rehabilitation Services, 2nd Floor 8-11 Queen Square, London, WC1N 3BG UK; 2 SLT Department, Imperial College Healthcare Trust, Charing Cross Hospital, London, UK; 3 Clinical Audiology, Speech and Language Research Centre, Queen Margaret University, Edinburgh, UK; 4 Swiss University of Speech and Language Sciences SHLR, Seminarstrasse 27, 9400 Rorschach, Switzerland; 5 Imaging Department, University College London Hospitals, London, UK; 6 Communication Sciences & Disorders Department, Syracuse University, Syracuse, NY USA; 7 Division of Psychology and Language Sciences, University College London, London, UK

**Keywords:** Ultrasound, Assessment, Dysphagia, Swallowing, Laryngeal function

## Abstract

Ultrasound (US) has an emerging evidence base for the assessment of swallowing and laryngeal function. Accessibility and technological advances support the use of US as a clinical assessment tool; however, there is insufficient evidence to support its translation into clinical practice. This study aimed to establish consensus on the priorities for translation of US into clinical practice for the assessment of swallowing and laryngeal function. Nominal Group Technique (NGT) was used as a formal method of consensus development. Clinicians and academics, all members of an international US working group, were invited to participate in the study. Two NGT meetings were held, where participants silently generated and then shared ideas. Participants anonymously ranked items. Rankings were aggregated before participants re-ranked items in order of priority. Discussions regarding rankings were recorded and transcribed to inform analysis. Member-checking with participants informed the final analysis. Participants (*n* = 15) were speech and language pathologists, physiotherapists and sonographers representing six countries. Fifteen items were identified and prioritised 1–13 (including two equally ranked items). Reliability, validity and normative data emerged as key areas for research while development of training protocols and engagement with stakeholders were considered vital to progressing US into practice. Analysis revealed common themes that might be addressed together in research, in addition to the ranked priority. A measured approach to the translation of US into clinical practice will enable effective implementation of this tool. Priorities may evolve as clinical and professional contexts shift, but this study provides a framework to advance research and clinical practice in this field.

**Supplementary Information:**

The online version contains supplementary material available at 10.1007/s00455-022-10413-9.

## Introduction

Ultrasound (US) has an emerging evidence base for the assessment of swallowing and laryngeal function [1]. Early research identified the ease with which anatomical landmarks and soft tissues of the upper aerodigestive tract could be identified using US [2]. Since then, US has been validated as a tool to assess the size and structure of the muscles involved in swallowing [3–5], offering an opportunity to assess impairment at an anatomical level. It has also been applied to the evaluation of swallowing and laryngeal kinematics, in particular the assessment of vocal fold movement [6, 7], tongue and posterior pharyngeal wall movement [8, 9], opening of the upper oesophageal sphincter [10], displacement of the hyoid [11–15] and thyrohyoid approximation [16, 17].

The advantages offered by ultrasound for the assessment of swallowing and laryngeal function include its accessibility, portability, lack of radiation and low levels of patient invasion which make it suitable for prolonged or repeat examinations, as well as hard-to-reach patient groups [18]. Recent research exploring manual versus automatic tracking of swallowing-related movements indicates potential for more efficient analysis of US images and improved clinical utility [19]. The emerging evidence that supports the use of US to detect bolus residue and aspiration may also extend its range of potential applications in the clinical setting [20–22]. Technological advances in US imaging quality [23], and increasing accessibility and portability of US devices [24, 25], further support the rationale to determine the optimal approaches for effective implementation of US into clinical practice that are sustainable and evidence-based.

The application of US as a diagnostic tool to guide therapeutic swallowing and laryngeal intervention remains underexplored. A recent rapid review in this area [1] indicated the need for further research prior to translation of US into clinical practice for this purpose. Other professional groups have started to translate US as a clinical evaluation tool [26–28] but a lack of sufficient evidence has hindered implementation [29]. Parallels can also be drawn within the dysphagia community when reflecting on the implementation of existing clinical instrumental evaluation tools, specifically Endoscopic Evaluation of the Larynx (EEL), Flexible Endoscopic Evaluation of Swallowing (FEES) and Videofluoroscopic Swallowing Studies (VFSS). Despite being the tool(s) of choice for dysphagia evaluation [30, 31], heterogeneity of protocols, outcome measures and lack of reliability have beleaguered clinical practice and impacted patient care [32]. More recent development of standardised and validated protocols for FEES and VFSS has enabled clinicians to become more effective in formulating clinical management plans [33–35]. A systematic approach to translation of US into dysphagia assessment practices acknowledges these limitations and will assist with the implementation of US in a logical and appropriate way.

### Translating US into Clinical Practice

Implementation science [36] represents one possible approach to facilitating integration of US into clinical practice. It offers a rigorous, structured and evidence-based methodology to implement a technique into regular use by clinicians, as well as taking into account elements that may limit its acceptance [37]. This balance between research and practice encourages the consideration of facilitators and mitigation of barriers which may be key to the implementation of US for the purpose of swallowing and laryngeal assessment. This is particularly relevant due to the complex and heterogeneous environments in which clinicians work, including different policy contexts, organisational systems, and professional domains.

Normalisation Process Theory (NPT) is a theory of implementation science, offering a structured approach to the normalisation of US as an evidence-based clinical assessment tool [38]. NPT considers clinical contexts, clinicians and practices to understand the translational gap between evidence, policy and practice, and is often used in healthcare settings [39, 40]. Prior to normalisation of US as an evidence-based clinical assessment tool, there are a broad range of topics (or items) that require further investigation [1, 18]. Even though many such understudied US topics have been identified, there is little guidance as to which are the most important or necessary. Without this information, the translational gap between evidence and practice cannot be understood. Consensus methods can help prioritise work to support systematic translation of US into clinical practice [41].

The development of prioritised agendas is highly relevant to healthcare research. The World Health Organization and groups such as the James Lind Alliance provide opportunities for patients, clinicians and researchers to come together to identify evidence uncertainties and generate research priorities across many aspects of healthcare [42, 43]. Prioritised agendas have been applied to the development of a research programme by people with aphasia [44] and the identification of a core outcome set for researchers working in this area [45]. Consensus-building informs research commissioning and ensures that funders are aware of the issues that matter most to the people to whom the research applies. A prioritised agenda enables us to do the “most good” with limited resources. It does not introduce new information but quantifies the level of uncertainty of the topics that are priority-ranked [46].

### The International US Working Group

In response to the translational gap between evidence and practice, a group of clinicians and researchers identified a need to develop a prioritised research agenda to support the translation of US into clinical practice for the assessment of swallowing and laryngeal function. This international working group formed in Spring 2020 when restrictions related to the novel coronavirus Sars-Cov-2 limited access to more established clinical imaging tools due to infection risk [47]. Members of the group have a mutual interest in the clinical utility of US for the assessment of swallowing and laryngeal function. The group’s objective is to motivate further research via collaboration between clinicians and researchers. They identified a need to undertake this consensus study to inform the prioritised research agenda for the implementation of US into clinical practice.

## Methods

### Study Design

To gain a comprehensive understanding of the relevant needs associated with translating US into clinical practice, a qualitative research method was identified as appropriate. Nominal Group Technique (NGT) is a structured method of generating ideas, devising solutions and producing recommendations for best practice [48, 49]. It encourages equal contribution from participants and enables collaboration between a mix of professional groups. It has been extensively reported in the healthcare literature as a method of synthesising expert opinion, particularly in the absence of published data relating to a subject [41, 50]. NGT has successfully been used to enable clinicians, researchers and patients to work collaboratively to develop a prioritised agenda to drive profession-specific research in health [51]. NGT methods were specifically selected to provide equity in participants’ contributions, enabling people to contribute in a structured and systematic way [49].

The study was given ethical approvals by the Human Ethics Committee, University of Canterbury, New Zealand (Ref HEC 2021/12/LR-PS). Work undertaken for this study has been reported using the Consolidated Criteria for Reporting Qualitative Research (COREQ) [52].

### Participants

A purposive sampling strategy was used, whereby participants were recruited from the international US working group of experts who represented multidisciplinary clinical professionals with an interest in US, and who identified the research gaps. The group was established in April 2020 and formed through practical and/or theoretical interest in US. The group comprises eighteen members from four professional groups and seven different countries. A core aim of the group has been to collaboratively advance an US prioritisation agenda with structure and rigour. All group members were invited verbally and via email to participate in the NGT process if they considered their own experience and ability to respond to the research question as appropriate. The invitation was not extended outside of the international group as the group size and range of expertise were considered sufficient to meet the research aims and requirements of NGT methodology. NGT participants provided written consent and demographic details pertaining to their profession and highest academic qualification, years of clinical and dysphagia practice, practical experience of US, patient cohort and country of work.

### Developing the Research Question

The rapid review [1] helped to identify broad target areas for future research. With this in mind, a subgroup of the international US group refined the research question to ensure it met the study aim and was clear and well-defined for the purpose of the NGT process [53].

Once the question had been agreed by the subgroup, it was refined by the wider membership and agreed on a-priori 1 month before embarking on the NGT process. The final question was sent out to participants 2 weeks before stage one of the NGT process. Participants were instructed to spend time thinking about the research question but not research or generate any written responses in advance of the meeting.

**Fig. 1:**
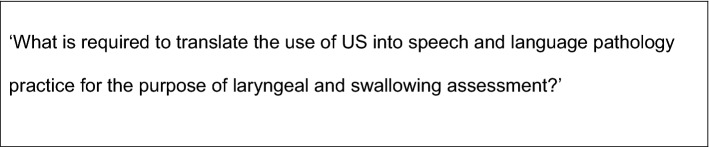
NGT research question

The question was designed to encompass both swallowing and laryngeal function as it was hypothesised that those areas share the same requirements for translation into clinical practice (Fig. 1).

### Nominal Group Technique Process

**Fig. 2:**
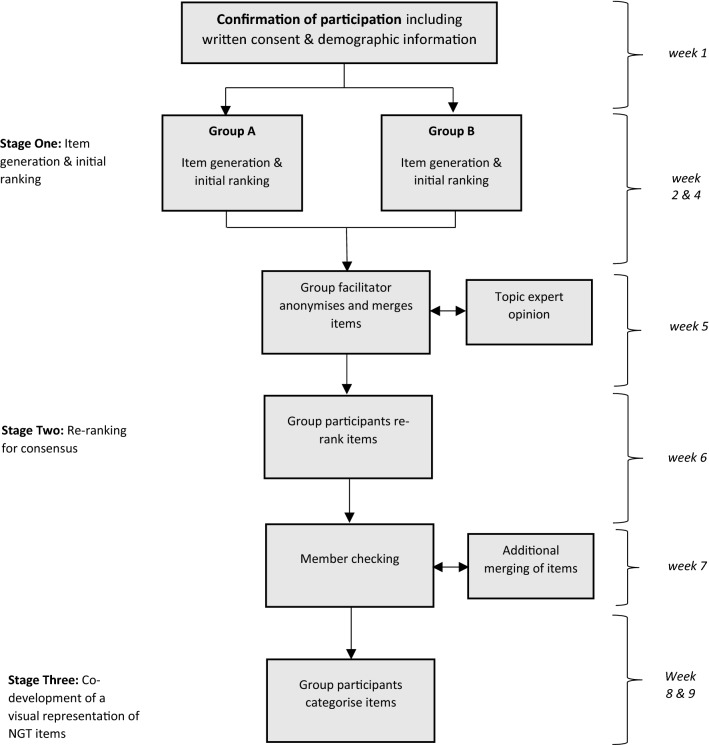
Nominal group technique process for US consensus recommendations

The NGT process comprised a two-stage ranking process commencing with a 90-minute online group session (stage one), followed by email consultation (stage two). Participants were divided into two groups (A & B) for stage one to allow people across different time zones to participate. Each meeting lasted 90 minutes and was held on a video conferencing platform (Zoom). Groups were facilitated by a researcher experienced in NGT processes in line with guidelines for conducting NGT meetings [54]. The central topic guide was agreed in advance by the research team (see supplementary file 1). All meetings were recorded. A third stage was added, whereby member checking included the co-development of a visual representation of the study results. Figure 2 provides an overview of the NGT process.

#### Stage one: Item Generation and Initial Ranking

At the start of the session, participants were reminded of the NGT process by the facilitator. The research question was presented (see Fig. 1) and participants were given 10 minutes to silently generate written ideas in response. In line with NGT methods, each participant in turn was asked to present an item from their list of ideas, using a ‘round robin’ approach, until all lists had been exhausted. Generated items were captured on a shared PowerPoint slide. Participants were then provided 10 minutes to silently identify and rank the eight items they considered most important by placing a number from 1 to 8 to reflect which is most important (8) and least important (1). Participants emailed their ranked items to the facilitator to maintain anonymity and reduce bias during the ranking process. Participants were invited to provide written justification for their ranking, plus any additional comments.

Following the online meeting, scores were aggregated by the facilitator to generate a list of items per group. The list provided information on scores and the group mean ranking for each item. As per guidelines, items describing the same ideas from the two groups were merged and scores combined, following discussion and agreement between two topic experts [54].

#### Stage Two: Re-Ranking to Generate Consensus Recommendations

The ranked list from stage one was emailed to all participants 5 days after the completion of stage one. Participants were asked to review the list and identify and rank the eight items they considered to be of most (8) to least (1) importance to the research question. To maintain anonymity within the international group, participants were asked to return their responses to the independent facilitator by email.

The facilitator again aggregated scores to generate the final priority list of the sum scores. The list was presented to the participants at a consequent group meeting for member checking [55]. These discussions were recorded and transcribed by the lead author for review. Any data relating to NGT rankings were extracted from the transcription and used to define each NGT item. Comments and reflections on the ranking process were also extracted. During this discussion participants identified items they felt had been represented more than once, but that represented the same concept. Participants discussed and unanimously agreed on the subsequent merging of items.

#### Stage Three: Co-Development of a Visual Representation of NGT Items

Items were grouped into categories and presented to NGT participants by the lead author. Six different diagrammatic representations demonstrating the relationships between items and their categories were also presented. All participants were invited to critique these diagrams and co-develop a final figure.

## Results

### Participants

Of the 18 members of the international group invited to participate in the study, 15 were able to take part. One did not feel they met the inclusion criteria and two withdrew from the study due to personal circumstances.

Group A comprised seven participants, all speech and language pathologists (SLPs). Group B comprised eight participants, six SLPs, one respiratory physiotherapist and one head and neck sonographer. Professional affiliation, country of practice and qualifications of the group are presented in Table 1.

**Table 1:** Demographics of NGT group (*n* = 15)

	Participants (*n* = 15)
Professional background	
SLP	13
Respiratory PT	1
H&N sonographer	1
Country of current practice	
England	9
Scotland	1
Netherlands	1
Switzerland	1
New Zealand	2
United States of America	1
Professional qualification	
PhD	5
MSc/MRes	5
PGDip	2
BSc	3

All but one participant specialised in adult care. Clinical experience of the group ranged from 6.5 to 41 years, median 16 years. Years of dysphagia practice among SLP participants ranged from 5 to 39 years, median 15 years. The sonographer and physiotherapist had no experience in dysphagia management. All but one member of the group had practical experience of US.

### NGT Rankings

In stage one, Group A generated 23 items and Group B 36 items for individual ranking. Following the ranking process and merging of similar items the initial list comprised 28 items. A list of all generated items from each group can be viewed in supplementary file 2.

After the re-ranking process in stage two, the list comprised a total of 21 items. Of these, 10 items were merged resulting in a final list of 15 items, ranked 1–13 with two items having received equal ranking. These final rankings are presented in Table 2.

**Table 2:** Items required to translate the use of US into SLP practice for the purpose of laryngeal and swallowing assessment

Rank	Item
1	Reliability
2	Normative values
3	Discriminant validity
4	Criterion validity
5	Training protocols and competencies
6	Measurement and interpretation
7	Standardised assessment protocols
8	Equipment specifications and settings
9	Understanding of swallowing movements in an US image
10	Evidence to support clinical utilisation
= 11	Resourcing
= 11	Infrastructure and ‘buy-in’
= 12	Recognition and guidance from professional bodies
= 12	Multi-professional input
13	Patient and public involvement

= represents items of equal ranking

The following descriptions summarise the discussions of study participants in relation to each item.

#### Reliability (Rank 1)

The highest ranked item related to the theme of reliability under which a range of items were raised, specifically, the establishment of test–retest reliability and inter- and intra-rater reliability in data acquisition including scanning, image selection and measurement. Participants noted the need for reliability among online and offline measurements as well as automated and manual measurements. Participants acknowledged that reliability may differ depending on the population sample and therefore expressed the need to establish reliability in both healthy and patient groups.

#### Normative Values (Rank 2)

Establishment of ‘normal’ values for the biomarkers representing swallowing and laryngeal function was described as requiring the acquisition and measurement of biomarkers in healthy participants to generate normative data. These data will need to capture the variation in normal function with age and across different swallowing conditions (e.g. bolus’ of different size and texture) and will serve as a reference value from which to judge disordered function. Participants recognised that some measurements may not be significantly different in patient and healthy populations, as normal values can have a broad distribution. The ability to detect differences between normal and disordered function also relates to discriminant validity (item 3). It was also acknowledged that, for patient groups, deviation from ‘normal’ values may carry greater clinical risk due to associated health issues. The importance of understanding the clinical significance of abnormalities was highlighted. Participants also commented on the potential for disconnect between measurements used for research versus clinical practice and noted the need for US measurements to be practical and achievable in the clinical setting.

#### Discriminant Validity (Rank 3)

Discriminant validity was defined as an understanding of the sensitivity and specificity of US measurements for detecting the conditions of concern, for example, the ability to accurately diagnose the presence versus absence of aspiration, disordered vocal fold movement or (subtype of) dysphagia. Participants also felt it important to understand the smallest unit of difference that US can measure to capture changes in swallowing or laryngeal function. These changes may relate to function pre- and post-intervention or progression of a condition over time. Participants recognised that the establishment of discriminant validity related to the reliability of image acquisition and measurement (item 1) and will also support the selection of biomarkers (item 2) for swallowing and laryngeal assessment. Discriminant validity will also help to define the selection of patient populations in which US assessment can be used. Participants also stated that establishment of discriminant validity will enable decisions about how US can be used in clinical practice, for example as a diagnostic, screening or monitoring tool.

#### Criterion Validity (Rank 4)

Criterion validity was defined as the need to understand if and to what extent US provides similar information to existing instruments of choice. The group agreed that the existing tools for comparison are VFSS and FEES for swallowing assessment and EEL for evaluation of laryngeal function.

#### Training Protocols and Competencies (Rank 5)

Several items were proposed for possible inclusion into training protocols, with the aim of ensuring safety and governance. These included:

- a statement on the operator’s scope of practice
- a description of the knowledge and skills required to perform US for the purpose of swallowing and laryngeal assessment
- minimum standards for competence
- guidance on who delivers the training (including the skill-level of the trainer and trainee)

Participants agreed that training should provide an understanding of US technology (see item nine) and the possibility of including material to support documentation of the above was also raised.

#### Measurement and Interpretation (Rank 6)

Current variation in approaches to measurement (and interpretation) challenge measurement reliability (item 1) and the establishment of standard measurement. The approach to measurement or interpretation depends on the purpose for which US is used. For example, muscle US requires different measurement approaches to those used for measurement of swallowing biomechanics or symptoms. Measurement and interpretation of images directly relates to discriminant validity (item 3). It was acknowledged that biomarkers (e.g. vocal fold or hyoid movement) could be interpreted by the operator without measurement; however, more refined assessment requires measurement. Even with measurement, the information will require contextualising and interpreting (see discussion under item 2). Participants suggested that practice should be standardised via the introduction of protocols, rating tools or measurement algorithms. Participants discussed that such protocols would make US time-efficient for clinical practice but acknowledged the need for further research in this area.

#### Standardised Assessment Protocols (Rank 7)

Standardised assessment protocols may include descriptions of patient criteria and rationale for the US assessment, as well as selection of equipment (see item 8). Specification of probe position and bolus types for assessment was also considered important, as was the need to specify the recording parameters for each aspect of swallowing or laryngeal function.

NGT participants recognised that the protocols for US assessment may differ according to the setting requirements (e.g. clinical versus research settings). As per item 2 (normative values), the need for a practical and accessible protocol in the clinical setting was highlighted, including the need for the protocol to be applicable to as many patient populations as possible.

#### Equipment Specification and Settings (Rank 8)

Specification of the minimum technical requirements and recommendations for sufficient US image quality was suggested as one possible approach to enabling US operators to make appropriate selection of US devices and settings. These specifications should include a description of which device, probe, and settings to use. There was acknowledgement that this would vary depending on the purpose of use. For example, assessment of muscle and soft tissue structures for swallowing will require a device where image optimisation could be turned off and probes could be hand-held or placed in a headset. The need for further research to understand and guide the practicalities of image set-up and acquisition (see item7) was acknowledged by participants.

#### Understanding of Swallowing Movements in an US Image (Rank 9)

To translate the use of US into clinical practice, there needs to be a greater understanding of how to read and interpret US images of head, neck and swallowing anatomy and physiology. There is overlap in scope between this and previous items, particularly training and competency development (item 5), since this item would need to include training to understand US technology, devices, and settings.

#### Evidence to support clinical utilisation (Rank 10)

Evidence to support the use of US in clinical practice for swallowing and laryngeal assessment is necessary. One suggestion for generation of such evidence included research focussed on clinical translation, such as understanding the clinical environment in which US can be used and the limitations and benefits of US when used for this purpose. Another suggestion included the development of a clinical database of images to enhance practice-based evidence. Participants also suggested development of a core outcome set to help establish the impact of clinical implementation, which would enable clinicians to assess the impact of introducing US within their service. Participants raised the need to keep up to date with the latest ‘good practice’ in those clinical specialities already using US to help inform implementation and innovation for this unique purpose.

#### Resourcing (Rank 11)

Resourcing includes support from managers and service providers, including health organisations (see item 12). Examples of resources generated by the group included equipment and consumables such as US devices, servicing contracts, physical space and funding. This item was ranked jointly with infrastructure and buy-in (below).

#### Infrastructure and Buy-In (Rank 11)

The specific requirement for buy-in from managers and commissioners was identified as a separate but equally ranked item to resources. Examples of ‘buy-in’ generated by participants included authorisation from the relevant clinical department to develop a business case for the use of US*.* This will also require implementation support and recognition within the wider organisation. Participants commented that research projects demonstrating the utility of US (item 10) will support business cases.

#### Recognition and Guidance from Professional Bodies (Rank 12)

The involvement of professional bodies to ensure that the extended scope of practice for operators using US for the purpose of swallowing and laryngeal assessment was recognised by participants and ranked in jointly with multi-professional input (below). This is consistent with the approach taken when other swallowing and laryngeal assessment tools, such as FEES and laryngoscopy, were adopted into clinical practice.

#### Multi-Professional Input (Rank 12)

The roles and responsibilities of different multidisciplinary professionals involved in using US to assess swallowing and laryngeal function was raised by participants as an item that needed elucidation to support the translation of US into clinical practice. Along with SLPs, participants defined other key professional groups as including engineers and radiologists. They also raised the need to decide whether US assessment can be conducted by uni-disciplinary professionals or required interdisciplinary skills. Participants highlighted that the level and combination of skill(s) required by the US operator needed to be determined to clarify the scope of practice for SLPs (see item 5).

#### Patient and Public Involvement (PPI) (Rank 13)

During the member checking process, it was agreed by consensus to use the INVOLVE definition of PPI [56].The statement focusses on the key values of PPI: respect, support, transparency, responsiveness, fairness of opportunity and accountability which were all considered foundational for the translation of US into clinical practice. The group acknowledged while PPI was ranked in the final position, this did not reflect the overarching necessity of patient-public engagement to the success of US implementation.

**Fig. 3:**
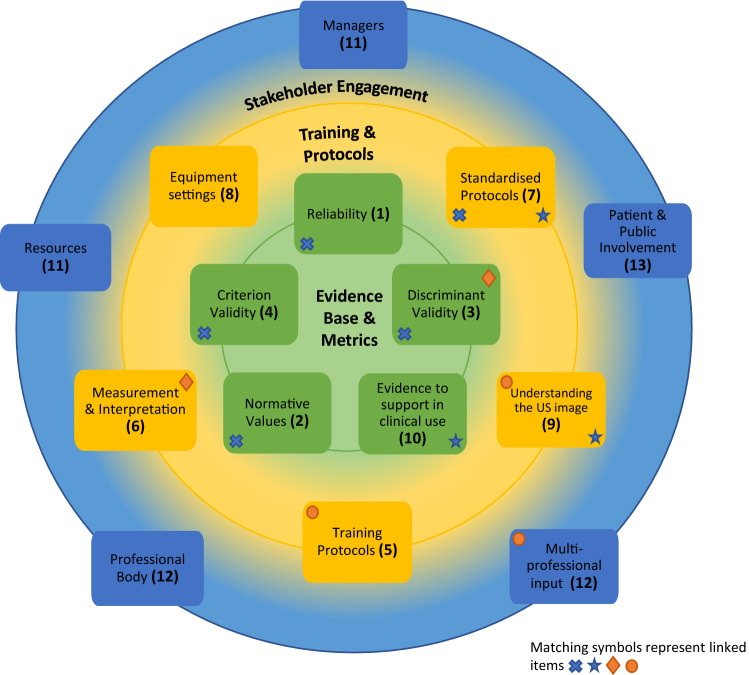
Visual representation of NGT results

Below is a representation of the results which depicts the overarching themes and relationships between NGT items. The co-developed figure represents the three main categories of work that the NGT items can be considered within. These categories are stakeholder engagement (blue band), training and protocols (yellow band), and evidence base and metrics (green band). Each item is represented by a rectangle within these categories. The shape(s) within the rectangle represent the relationships between items. The cross represents links between reliability, discriminant validity, criterion validity and standardised protocols. The diamond represents the links between discriminant validity and measurement and interpretation. The star represents links between standardised protocols and evidence to support clinical use of US. The circle represents the links between training protocols, understanding the US and multi-professional input. Numbers in brackets represent the original ranking of each item (Fig. 3).

## Discussion

The aim of this study was to identify the elements required to translate the use of US into clinical practice for the purpose of laryngeal and swallowing assessment. Participants identified a high-priority need to establish reliability and validity of US data acquisition and measurement, plus establish normative values to serve as a reference from which to diagnose disordered function. These items are not stand-alone and relationships between items are not linear. For example, reliability and validity of laryngeal and swallowing assessment may be viewed as interdependent issues. While US data acquisition may be reliable without being valid, validity requires that measures are acquired consistently [57–59]. Thus, studies may explore both validity and reliability at the same time. Further, reliable and valid assessment is related to assessment protocols and equipment selection; for example, reliability and validity may be impacted by the choice of US device. While there are some data to suggest that standard-sized US equipment may provide reliable and valid swallowing measures [5, 12, 16], this could not be confirmed for pocket-sized technology [24, 60]. The method of data acquisition, such as the use of hand-held versus fixed transducers, may further affect reliability [61, 62]; as might variability between different data collection settings, for example, data collected in the clinical versus research environment. The relationships between reliability, validity and the assessment protocol are reciprocal. An increased understanding of both reliability and validity is required to clarify which data acquisition procedures are most accurate for the assessment of laryngeal function and swallowing parameters. This needs to be combined with robust reproducibility between operators to enable clinicians to use US accurately and consistently.

The necessity for adequate reliability also relates to discriminant validity. For example, binary assessment of vocal fold function (i.e. presence versus absence of movement) is most often used to diagnose vocal fold palsy [1, 6]. When a linear probe with an appropriate range of frequencies is used, US shows reasonable sensitivity and good specificity for this purpose. However, binary assessment is not sufficient to develop tailored swallowing or voice therapy programmes. To derive meaning from the assessment of swallowing or laryngeal function, a greater range of quantitative information is required. Potential biomarkers for vocal fold movement include measurement of the inter-arytenoid distance, the glottic area, or glottic angle at the point of full vocal fold abduction and adduction [63, 64]. While preliminary data exist for the healthy pediatric and young adult (< 30 years) populations with respect to muscle thickness and echo intensity of the submental and tongue muscles [3, 65], and hyoid bone movement in healthy young [66] and ageing adults [15], no normative data exist to support the assessment using the vocal fold biomarkers described above. An essential requirement for using US as a diagnostic, rather than a screening or biofeedback tool, is its ability to discriminate between clinically significant changes. Discriminant validity is therefore important to define the boundaries for reliability and relies on the existence of normative data for comparison.

A link between reliability assessment and development of training protocols was also identified as part of the NGT process. Intra- and inter-operator reliability is dependent on the expertise and experience of the US operator. Operator training has shown to improve the reliability in the measurement of static images [67] and an association between reliability and operator skills has been documented for procedures including VFSS [68], high-resolution manometry [69] and muscle US data acquisition by physiotherapists [70]. The relationship between reliability and training is, however, reciprocal, and reliability data are required to determine the necessity and content of training protocols [60].

### Translating NGT Items into Clinical Practice

The items in this study represent priorities related to both primary research and clinical practice. This ‘bench to bedside’ continuum provoked debate among NGT participants and discussions ensued as to whether the research and clinical practice components should be separated. The research question was developed with the expectation that ideas would be generated across the research and clinical continuum, therefore a decision was made to preserve the original rankings since this is a true representation of the translational research continuum [71]. A close relationship between clinicians and researchers has proven to enhance the potential for translation into practice [72]; however, implementation needs to be robust and systematic.

Implementation science methodology, such as NPT, offers a structured approach to the normalisation of US as an evidence-based clinical assessment tool [73] and is a feasible next step towards progressing US as a tool for clinical swallowing and laryngeal assessment. To be successful, it will require a thorough evaluation of the barriers and enablers [74] but the grounding provided by this priority-setting exercise gives clear direction towards the next steps in the process. In the context of NPT, the NGT prioritisation exercise fulfils the initial step of sense-making (coherence) [38]. The NGT exercise and previous rapid review have laid the groundwork in defining the intervention (US) and the context (clinical practice) where implementation is required.

A practical way of progressing the implementation of US using the NPT approach would be to begin training cohorts of SLPs in the applied aspects of US technology to acquire images of anatomical structures in the head and neck. This would allow SLPs to develop practical skills (item 5) without focussing on the more complex competencies of biomechanical assessment and diagnosis while capturing practice-based data to fulfil items 1–4 of the NGT process. Qualitative research such as focus groups or interviews could be conducted in parallel with clinician-training to determine the opinions of dysphagia practitioners and other stakeholders about the barriers and facilitators to using US in clinical practice. This process of action, alongside in-depth appraisal of the intervention would allow the “implementation potential” of US to be fully explored and maximise its chances for successful translation into practice to benefit clinicians, researchers and patients [38].

NPT offers an alternative approach to implementation used by other professional groups such as physiotherapists who have taken a more applied approach to the implementation of US for clinical assessment purposes. For example, the focus in thoracic US has been to develop practice-based evidence, prior to the advancement of psychometric data. This has led to enthusiastic adoption of US into critical care and respiratory physiotherapy services [75–77]; however, the lack of formal research evidence is now considered a barrier to implementation and acceptance of US for this purpose [29]. There is an opportunity to avoid this conflict by implementing swallowing and laryngeal US into clinical practice in a systematic and evidence-based way, using lessons learned from other professional groups and previous implementation of assessment tools in the SLP profession.

The implementation process can be expedited using the knowledge and understanding gleaned from this study. More specifically, the work needed to address the ranked items in this study does not always need to be undertaken in chronological order. Completing aspects either in parallel or via a cyclical approach will advance the translation of US into practice as quickly as practicable. For example, results emphasise the importance of having assessment and analysis protocols (items 6–8), particularly for equipment and settings. Establishing such procedural aspects will, in turn, improve inter-and intra-operator reliability (item 1) [78, 79]. A better understanding of the clinical environment in which US will be used (item 10) will also facilitate an understanding of gaps that may exist between reliable assessment in the research setting versus the clinical setting. This will support the development of clinically realistic and achievable protocols (items 6 & 7). Implementation of US as a swallowing or laryngeal biofeedback (therapy) tool may mitigate the requirement for diagnostic values [80]. This alternative use may also enable US to be transitioned into clinical practice sooner, while primary research continues. The precedent for this has already been established in the field of US and developmental speech disorders [81].

### Study Limitations

There is an inherent risk of bias in any priority-setting process driven by expert consensus. Study participants had differing levels of familiarity and experience which could have led to, for example, bias due to more experienced experts taking the lead in priority setting [82]. The use of NGT methodology minimised bias that would have arisen from a less structured approach and has enabled efficient and rigorous development of a prioritised agenda [83].To minimise facilitator-driven bias, an NGT facilitator with no prior knowledge of the US topic was recruited. This lack of topic knowledge resulted in NGT participants merging items from groups one and two after stage one of the NGT process. Bias was minimised via agreement of merged items with a second participant. The facilitator did not take field notes during the NGT process though evidence to support understanding was provided by meeting transcripts and email responses from participants to the facilitator. Member-checking enabled greater richness of perspectives and ensured that results reflected participants’ views as closely as possible. Participants were only recruited from the international working group. Since the inception of this group, members have been recruited via a snowball approach to ensure wide representation. The inclusion of an experienced head and neck sonographer and US physiotherapist ensured the inclusion of perspectives from non-dysphagia trained professionals with more advanced skills in US practice. It is possible that representation from dysphagia-practitioners outside of SLP, such as gastroenterologists and otolaryngologists, may have generated additional items and increased the applicability of this study.

It is not the remit of NGT methodology to benchmark ranked elements with the existing evidence base. Reviews of current evidence are readily available in the published literature [1, 18, 84]. The decision to combine the assessment of swallowing with laryngeal function was based on the premise that the elements required to translate this assessment tool into clinical practice for both purposes would be comparable. Assessment of swallowing using endoluminal sonography was not included. The evidence base for each ranked item may diverge depending on the exact purpose of use. This includes the use for non-assessment purposes, such as for education or biofeedback. Further work is required to align the ranked elements with the existing evidence base to establish the gaps in the literature for each item.

### Future Directions

The interest in US as a swallowing and laryngeal assessment tool has gathered momentum in the past 18 months [1, 18]. This could lead to rapid application of US into clinical practice without developing the requisite evidence base and therefore compromise patient care. The findings of this study will equip clinicians and researchers with the knowledge and understanding to support a measured approach to the implementation of US for clinical assessment of swallowing and laryngeal function with efficacy and sustainability.

There is a need for primary research in the validation and reliability of US for assessment purposes which can be conjointly addressed with a practical, skill-based approach to implementation to support the development of assessment protocols and training packages. While these priorities may evolve as clinical and professional contexts shift, this study provides the context for further swallowing and laryngeal-focussed US research and clinical activity in this field.

## Supplementary Information

Below is the link to the electronic supplementary material.

Supplementary file1 (PDF 151 kb)

Supplementary file2 (PDF 113 kb)
