# Supplementary material for: Translating Ultrasound into Clinical Practice for the Assessment of Swallowing and Laryngeal Function: A Speech and Language Pathology-Led Consensus Study
Source: Dysphagia. 2022 Feb 24;37(6):1586–98. doi: 10.1007/s00455-022-10413-9 (PMC8867131; doi:10.1007/s00455-022-10413-9)
Supplement: Supplementary file 1 — Supplementary file1 (PDF 151 kb) [file 455_2022_10413_MOESM1_ESM.pdf]

Supplementary File 1: Translation of Ultrasound into Clinical Practice for the Assessment of Swallowing and Laryngeal Function - Central Topic Guide

Facilitator: AV

This topic guide has been designed, reviewed, and reformulated by the research team for the project and should be used in conjunction with slides prepared for session.

Duration of NGT meetings: 90 minutes

1. Introduction of group facilitator and acknowledge that group members are all known to one another. Final check that consent paperwork has been completed.

2. Start video recording

3. Outline agenda for meeting:
   Background and introduction to NGT
   Introduction to research question
   Idea generation
   Discussion
   First round of ranking
   Finish

4. Provide background overview to meeting summarising (JA as project lead):
   The group had Identified the need to develop a consensus statement on the translation of ultrasound into SLT practice to support prioritization of future projects & research in this area (inside & outside this group)
   Considered different methodologies to do this
   Agreed NGT
   Split into time zones to realistically achieve this task

5. Remind group of objectives of the NGT process:
   Assure different processes for each phase of creativity
   Balance participation among members
   Incorporate mathematical voting techniques in the aggregation of group judgment

6. Introduce research question: What is required to translate the use of ultrasound into SLP practice for the purpose of laryngeal and swallowing assessment?

   (Items 1-6 = 30 minutes)

7. 10 minutes silent idea generation

8. Invite participants to share ideas in round robin fashion until no further ideas remain. During the process facilitator to ask for clarification, using prompts such as:
   "Can you explain how x is different to y"
   "Can you provide an example to make this clearer"
   "Can you provide further information to explain what this means"
   (30 minutes)

9. 10 minutes silent rating- facilitator to invite participants to identify top 8 most important ideas from list and consequently rank these from 0-8, (8= most important, 1=least important)

10. Invite participants to share ranking via email with facilitator

11. Close and thank you. Advise group on next steps.

(Items 10-11 = 10 minutes)
