# Supplementary material for: Translating Ultrasound into Clinical Practice for the Assessment of Swallowing and Laryngeal Function: A Speech and Language Pathology-Led Consensus Study
Source: Dysphagia. 2022 Feb 24;37(6):1586–98. doi: 10.1007/s00455-022-10413-9 (PMC8867131; doi:10.1007/s00455-022-10413-9)
Supplement: Supplementary file 2 — Supplementary file2 (PDF 113 kb) [file 455_2022_10413_MOESM2_ESM.pdf]

Supplementary file 2: Translation of Ultrasound into Clinical Practice for the Assessment of Swallowing and Laryngeal Function - List of all NGT items generated by Group A and Group B

**Group A**
• Clinical valid and meaningful measurements (meaningful includes reliability, normative values that are achievable in clinical practice – with normative values- for ages/diff patient groups etc)<br>• Training protocols and competencies- who delivers this, what's the right skill level of the SLT before they start training (who, what, why and when)<br>• Minimal technical requirements for sufficient image quality<br>• Analysis routines or algorithm or protocol to analyse the images or the ultrasound videos and to make it time efficient you need an algorithm to analyse the videos (dependent on purpose of use of US in SLT ax – because the aim is quite broad, as we can visualise muscles/structures/residue)<br>• Access to current gold standard to compare your US results to it.<br>• Understanding of the swallowing movements in US images – reading US anatomy, reading it and knowing what movement we see in a normal swallow.<br>• A definition of which US device, probe and settings, which will depend on what we are using it for (could same as min tech requirements for sufficient image quality)<br>• For clinical practice- ax protocols (position/direction/bolus/recording etc), at least descriptions of patient criteria/rationale for use/requirements for clinical setting versus research setting to make it practical and accessible<br>• Better understanding of what US is and how it works- what it is and why is shows this type of images<br>• What is the perception of using US in SLT as a clinical tool – what are the barriers to using US.<br>• Validity data – does US provide similar info to VDF and need data in terms of sensitivity and specificity as a diagnostic tool, no only correlation but do they agree.<br>• For visualising structures and muscles- need an US device that can turn off image optimisation<br>• A trouble-shooting, reflective peer support network<br>• What are we using as a biomarker for analysing swallowing – we are using hyoid at the moment, but what else can we use- we need a better understanding of normal variations.<br>• Standardised scoring or analysis system (falls into algorithm)<br>• Get buy-in from managers and service-providers – how do we do this. Develop research to demonstrate its worth. But need someone to pay for it.

- Reliability – intra and inter test reliability, acquisition-scanning image selection and measurement (diff levels of reliability we need to understand) does training improve reliability, what it the min difference that US can detect in terms of change to inform whether it can be used as diagnostic tool or screening tool or biofeedback
- Generalisability – are we going to be able to generalise across patient groups/check movement as patients get worse or better/is it applicable across patient groups
- Need data on normal swallow of US so we compare disordered swallow or normal swallow
- Clinical database to continue practice-based evidence
- Descriptions of measurement techniques inc reliability data for those techniques
- Research and development support
- Do we need a radiologist/another professional alongside the SLT to do US

Group B

- Standardised norms of swallow and laryngeal assessment in the normal population
- A formalised training programme for staff to learn
- Hypothesis that ultrasound has applicability to a wide range of aetiologies- Data on understanding utility in different clinical populations
- We need some standardised protocols on how to perform the various examinations that we want to do what images to take, what actions to capture on your examination.
- Specifying that we need to provide guidelines for US setting that are appropriate for various structures we are imaging
- To establish test–retest intra and inter reliability.
- Data acquisition, data measurement, online analysis and analysis of imaging
- Some agreement on how to interpret the examinations- a standard rating tool or various rating tools.
- Validity or correlation against a gold standard.
- To determine what are the key variables (most important) for a clinical assessment of laryngeal and swallowing skills on ultrasound.
- Multi-professional input- SLT, engineering, radiology, ENT, sonographers, PT.
- Recognition/guidance of the tool from relevant professional bodies to ensure extended scope of practice is recognised.
- Resources need to be available (need an US machine and a space that you can use) within local trusts/health organisations to implement- contracting for equipment/servicing/consumables/procurement.
- Funding.
- Authorisation from departments that your HoD is happy for you to implement the services- that they are supportive and aware.
- Raise awareness- BUT being clear with SLTs what the limitations and benefits of the tool are
- Influencers or prominent advocates- we need them
- Enthusiasm/interest/buy in from the SLT/P profession to take on this role and understand the added benefit
- Discriminant validity of ultrasound- what is the sensitivity, specificity of it for detecting the conditions that we are concerned about.

- Agreed documented competencies on knowledge and skills required to perform ultrasound for these purposes- so that we have governance and safety (minimum standards, reduce risk) (infection control).
- To develop a corpus of example images- ideally that would include normal and abnormal examples. For the images to also demonstrate the measurement of the key variables.
- Need more research/trying out to help guide practicalities for set up and image acquisition.
- Establishing professional networks for supervision/support.
- Within local governance- thinking about how images will be stored.
- Evidence around clinical utility- research re the clinical environment- clear questions
- We need to develop a patient pathway- referral processes for an ultrasound scan, appointment process, how to give results to patients- administrative support etc.
- Development of an outcome set to facilitate audit of impact of implementation in their service.
- Exploration of variation in patient performance across swallows or assessments.
- Linking to latest good practice in related US fields- keeping an eye on latest innovations in the field to inform us. What's hot and does it help us. Tapping into already established training resources and people and linking in with other professional bodies- who can support us with this.
- Understanding of patient selection for procedure- who is appropriate for which US assessment
- PPI (Patient Public Involvement)
- Consultant radiologist- for second opinions/support/advice/recommendations for other testing
- Equipment specification- variability across platforms- what are we recommending we use.
- Determine scope of practice for SLT/Ps- who would be other relevant professionals depending on patient complaint (roles and responsibilities of different professionals)
- Position statement/clinical consensus statement from this group.
- Understanding the potential use of US as a biofeedback tool
